# ^18^F-FDG gallbladder uptake: observation from a total-body PET/CT scanner

**DOI:** 10.1186/s12880-022-00957-5

**Published:** 2023-01-10

**Authors:** Anna Calabro’, Yasser G. Abdelhafez, Elizabeth K. A. Triumbari, Benjamin A. Spencer, Moon S. Chen, Domenico Albano, Christopher R. Cassim, Francesco Bertagna, Francesco Dondi, Simon R. Cherry, Ramsey D. Badawi, Fatma Sen, Lorenzo Nardo

**Affiliations:** 1 Department of Radiology, EXPLORER Molecular Imaging Center, University of California, Davis, 3195 Folsom Blvd, Davis, Sacramento, CA 95816 USA; 2 Nuclear Medicine Unit, South Egypt Cancer Institute, Assiut University, Asyut, Egypt; 3 Nuclear Medicine Unit, TracerGLab, Fondazione Policlinico Universitario A. Gemelli IRCCS, Rome, Italy; 4 Department of Biomedical Engineering, University of California Davis, Davis, CA USA; 5 Department of Internal Medicine, University of California Davis, Davis, CA USA; 6 Nuclear Medicine Department, University of Brescia and ASST Spedali Civili di Brescia, Brescia, Italy; 7 Department of Radiology, Sangre Grande Hospital, Eastern Regional Health Authority, Sangre Grande, Trinidad and Tobago

**Keywords:** Total-body PET/CT, Fluorodeoxyglucose F18, Positron emission tomography computed tomography, Gallbladder, Sodium-glucose transporter 1

## Abstract

**Background:**

Total-body positron emission tomography/computed tomography (PET/CT) scanners are characterized by higher signal collection efficiency and greater spatial resolution compared to conventional scanners, allowing for delayed imaging and improved image quality. These advantages may also lead to better detection of physiological processes that diagnostic imaging professionals should be aware of. The gallbladder (GB) is not usually visualized as an ^18^F-2-fluorodeoxyglucose (^18^F-FDG)-avid structure in routine clinical PET/CT studies; however, with the total-body PET/CT, we have been increasingly visualizing GB activity without it being involved in an inflammatory or neoplastic process. The aim of this study was to report visualization rates and characteristics of GB ^18^F-FDG uptake observed in both healthy and oncological subjects scanned on a total-body PET/CT system.

**Materials and methods:**

Scans from 73 participants (48 healthy and 25 with newly diagnosed lymphoma) who underwent ^18^F-FDG total-body PET/CT were retrospectively reviewed. Subjects were scanned at multiple timepoints up to 3 h post-injection. Gallbladder ^18^F-FDG activity was graded using liver uptake as a reference, and the pattern was qualified as present in the wall, lumen, or both. Participants’ characteristics, such as age, sex, body-mass index, blood glucose, and other clinical parameters, were collected to assess for any significant correlation with GB ^18^F-FDG uptake.

**Results:**

All 73 subjects showed GB uptake at one or more imaging timepoints. An increase in uptake intensity overtime was observed up until the 180-min scan, and the visualization rate of GB ^18^F-FDG uptake was 100% in the 120- and 180-min post-injection scans. GB wall uptake was detected in a significant number of patients (44/73, 60%), especially at early timepoint scans, whereas luminal activity was detected in 71/73 (97%) subjects, especially at later timepoint scans. No significant correlation was found between GB uptake intensity/pattern and subjects’ characteristics.

**Conclusion:**

The consistent observation of GB ^18^F-FDG uptake recorded in this study in healthy participants and subjects with a new oncological diagnosis indicates that this is a normal physiologic finding rather than representing an exception.

## Introduction

The gallbladder (GB) is not usually visualized as an ^18^F-fluorodeoxyglucose (^18^F-FDG)-avid structure in routine clinical positron emission tomography/computed tomography (PET/CT) studies, unless affected by an inflammatory or neoplastic process [1, 2]. Incidental non-pathologic ^18^F-FDG activity within the GB has been described in the literature in a limited number of case reports [3, 4] and a few retrospective studies [5, 6], mainly in patients who were diagnosed with cancer, as a phenomenon in which ^18^F-FDG accumulation within the GB is thought to be due to ^18^F-FDG excretion into the bile. For example, Murata et al. [6] retrospectively assessed a small cohort of cancer patients using a dual time point protocol on a conventional PET/CT scanner. A pattern of increasing GB uptake over time was detected. The other study [5] retrospectively assessed a large cohort of oncologic subjects using a conventional PET/CT scanner and reported an incidence of radiotracer accumulation within the GB of less than 1% and a direct correlation between ^18^F-FDG activity in the GB lumen and blood glucose levels. None of these studies specifically analyzed GB uptake in healthy subjects.

Recent improvements in PET instrumentation have led to long-axial field-of-view (LAFOV) and total-body PET/CT scanners [7]. These scanners are characterized by higher signal collection efficiency compared to the conventional PET scanners, which typically have an axial field-of-view of 20–30 cm. In addition, the total-body PET/CT scanner installed at our institution has an increased spatial resolution of ~ 3.0 mm that, coupled with the increase signal collection efficiency, allows for delayed imaging with improved image quality [8].

Delayed imaging protocols have been shown by several oncologic studies to better detect tumors based on different tracer kinetics between physiologic, benign, and malignant structures [9–11]. At our institution, we routinely scan at 2-h post-injection for oncological applications [12–14]. The gallbladder is frequently visualized during our routine clinical readings of oncological ^18^F-FDG total-body PET/CT studies. This observation, combined with lack of reported data on frequent GB visualization in healthy individuals, encouraged us to investigate GB ^18^F-FDG uptake in represented healthy volunteers and oncological patients.

## Materials and methods

### Study participants

After obtaining Institutional Review Board (IRB) approval (IRB #1341792, #1714742 and # 1470016) all participants gave written informed consent prior to being scanned.

This was a retrospective analysis of prospectively collected data for other research purposes. Data from two adult cohorts (healthy cohort and newly diagnosed lymphoma cohort), who underwent ^18^F-FDG PET/CT imaging on the uEXPLORER (United Imaging Healthcare, Shanghai, China) between June 2019 and January 2022, were analyzed. The first cohort included 48 healthy volunteers, without history of cancer or myocardial infarction in the past 5 years. This cohort included 3 subgroups, according to the injected ^18^F-FDG activity and racial/ethnic background (Table 1). The second cohort included 25 patients with an established diagnosis of lymphoma who were referred for a staging ^18^F-FDG PET/CT, before receiving any treatment.

**Table 1:** Details of Study Subgroups and Imaging Protocols

Characteristic	Healthy			Lymphoma
	Standard dose	Low dose	Standard dose^a^	
Participants	15	15	18	25
*Injected dose (MBq)*				
Mean ± SD (min–Max)	372 ± 17 (337–394)	20 ± 2 (17–24)	353 ± 37 (228–395)	292 ± 37 (197–328)
Number of scanning timepoints	6	3	3	2
1-h Dynamic scanning	Yes	Yes	Yes	No
Scan timepoints after injection (min) ^b^	40, 90, 180	40, 90, 180	40, 90, 120	60, 120 ^c^
CT tube current for each scan (mAs)	5, 50, 5, 5, 5, 5	5, 50, 5	5, 50, 5	50, 5

^a^Healthy participants from racial/ethnic minority population

^b^Images at the 40-min timepoint in healthy subgroups were extracted from a dynamic 60-min scan (i.e., the last 20 min were reconstructed)

^c^One out of the 25 patients was scanned at 90 and 120 min

Exclusion criteria for all groups were as follows: history of cholecystectomy, any known concomitant acute infection on the day of the scan, chemotherapy and/or radiation therapy in the past 3 years, major surgery within the last 6 months, pregnancy or breast-feeding, uncontrolled diabetes, and reported history of claustrophobia.

### Protocol and technical acquisition details

All subjects’ preparation included a minimum 6-h fasting period before ^18^F-FDG administration, with only water intake being allowed, and no exercise for at least 24 h prior to the scan.

All participants had their blood glucose levels measured before ^18^F-FDG injection. They were all manually injected through an i.v. peripheral access (antecubital vein in 67 and hand veins in 6 subjects).

Table 1 reports the methods, imaging protocol specifics and scan timepoints for each group. PET/CT acquisitions involved a low-dose or ultra-low-dose CT scan followed by PET acquisition. The tube current of all CT scans was automodulated by means of the manufacturer’s software. Details of employed CT tube current are summarized in Table 1. Tube voltage was 140 kV for all CT acquisitions. The estimated effective dose was 10 mSv for low-dose and 1 mSv for the ultra-low-dose CT acquisitions.

For the healthy cohort, PET acquisitions started immediately with the tracer injection and continued for 60 min (dynamic acquisition). The last 20 min (i.e., 40–60 min post-injection) of this acquisition were reconstructed for reviewing. The 60-min dynamic scan was followed by two more 20-min static acquisitions, at 90, and 120 or 180 min post-injection. For the lymphoma cohort, two static 20-min PET acquisitions were obtained at 60 min and 120 min post-injection.

### Image reconstruction and analysis

#### PET imaging

PET data were reconstructed using the manufacturer-provided software employing the 3D ordered subset expectation maximization (OSEM) algorithm with 4 iterations and 20 subsets into a 256 × 256 matrix using an isotropic voxel size of 2.344 mm. All the standard corrections were applied.

**Fig. 1:**
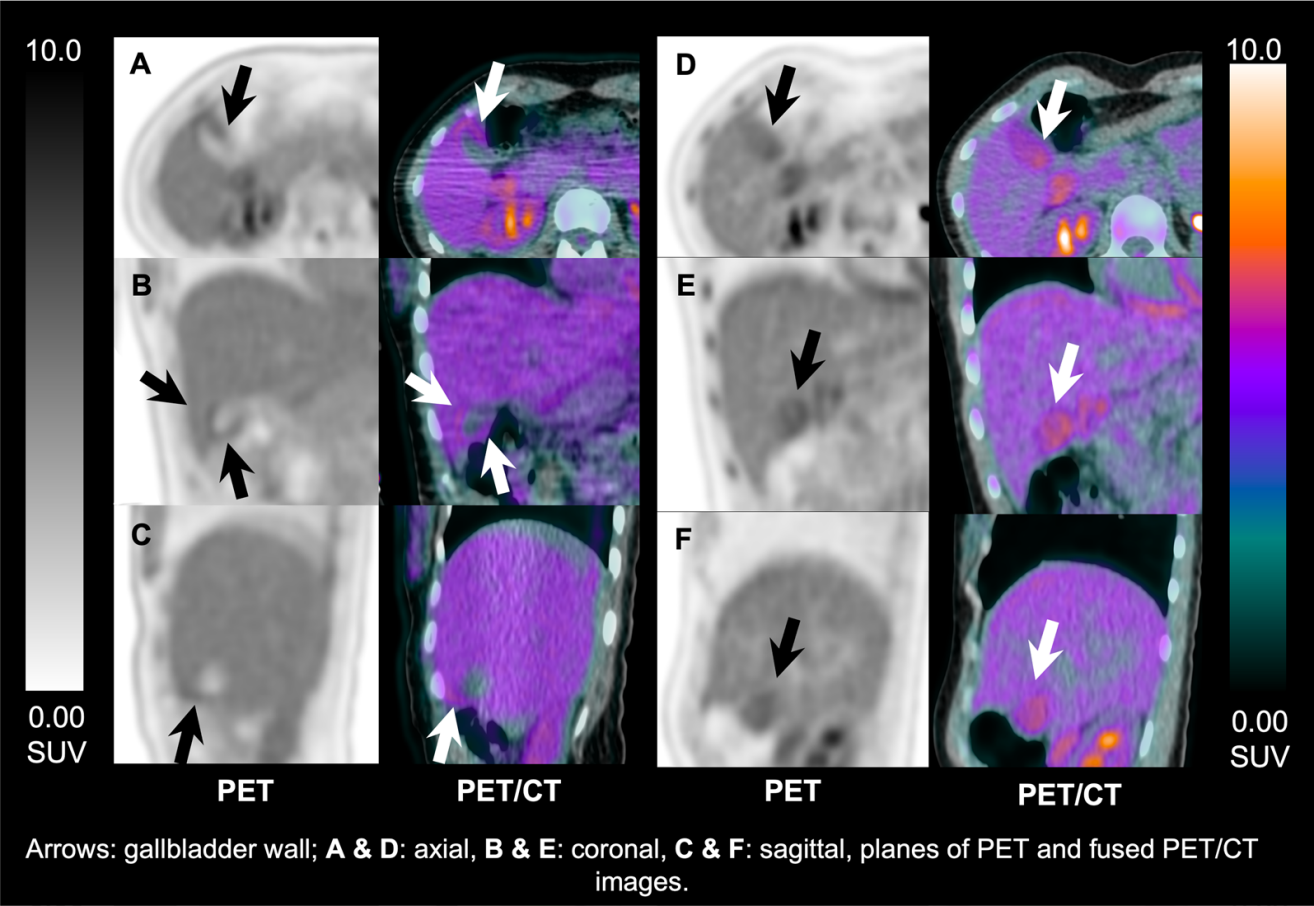
^18^F-FDG uptake patterns in the gallbladder wall and lumen. PET and fused PET/CT images after i.v. injection of 379 MBq in a 30 y/o healthy participant. **A**–**C** 40 min post injection scan shows gallbladder wall activity, equal to the liver; **D**–**F** 180 min post injection scan shows gallbladder luminal activity, higher than the liver

DICOM images were transferred to a viewing workstation running OsiriX MD (Pixmeo SARL, Bernex, Switzerland). Image series were displayed as 2D orthogonal views, including CT only, PET only, and fused PET/CT images. A team of three physicians, included 2 nuclear medicine physicians and a 3^rd^ year nuclear medicine resident, reviewed all images in consensus. Image assessment included visual analysis of the gallbladder ^18^F-FDG uptake. For each timepoint, uptake was recorded as either present or not present. When activity was present, further grading of the intensity level with respect to liver ^18^F-FDG uptake was defined as: gallbladder activity less than the liver, equal to the liver, or higher than the liver. The pattern of ^18^F-FDG uptake was also assessed as involving wall, lumen or both areas (Fig. 1).

A 3D volume of interest (VOI) was manually placed over the most intense area within the GB and the maximum standardized uptake value (SUVmax) was recorded for each timepoint. A large (~ 5–10 ml) VOI was placed on the right hepatic lobe at the level of portal vein and the mean value of SUV within this region was recorded and used as a reference. Scans with significant mis-registration between PET and CT data, e.g., as a result of respiratory motion, were excluded from quantitative analyses.

### CT data

All CT images were reconstructed using the manufacturer-provided software with a slice thickness of 2.344 mm to match those of PET and an in-plane voxel size of ~ 0.49 × 0.49 mm.

Low-dose CT images were also visually assessed for presence of gallstones and biliary sludge. Additionally, a quantitative assessment of hepatic steatosis was made. For each patient, volumes of interest (VOIs) were drawn on the low-dose CT portion of the scan in the following way: three 20-mm diameter spherical VOIs in the liver: one on segment II/III at the level of the portal vein, one on segment VIII and one on segment VII, avoiding inclusion of prominent vascular structures; three 20-mm diameter spherical VOIs were drawn on the spleen, aiming to sample 3 different areas of the organ. Mean Hounsfield Unit (HU) values in each VOI were recorded. Hepatic steatosis was defined by the presence of relative hypoattenuation of the liver at least 10 HU less than that of the spleen [15, 16].

### Data analysis

Data were summarized as frequency (percentages) or mean (standard deviation). Differences in qualitative features were compared using the chi-squared test or Fisher’s exact test, as appropriate. Differences in continuous data (e.g., age, body-mass index [BMI]) were compared between groups using Mann–Whitney U test. A two tailed *p* value < 0.05 was considered significant.

## Results

### Gallbladder visualization

Clinical characteristics of the study participants are summarized in Table 2.

**Table 2:** Clinical characteristics of the study participants

Characteristic	Healthy (N = 48)	Lymphoma (N = 25)
Age (years)	49.1 ± 12.9 (26–78)	55.8 ± 18.6 (24–83)
Weight (kg)	78.6 ± 16 (47.7–112.5)	80.8 ± 18.9 (55–126)
Height (cm)	169.7 ± 8.8 (152.4–195.6)	168.3 ± 9.5 (149.9–188)
BMI (kg/m^2^)	27.3 ± 5 (17.5–37)	28.5 ± 6.1 (18.4–44.4)
Blood glucose (mg/dl)	92.1 ± 13.4 (62–130)	97.4 ± 23.1 (66–156)
Fasting period (h)	10.6 ± 2.8 (6–16)	11.2 ± 3.8 (6–18)
Liver-Spleen (HU)	10.1 ± 7.7 (-5.1–21.1)	6.9 ± 10.9 (-31.1–17.7)
*Sex*		
F	28	14
M	20	11
*Race*^*a*^		
White	24	17
Black or African American	6	1
Asian	5	1
American Indian or Alaska Native	2	0
More than one race	0	1
Other	0	4
*Ethnicity*^*a*^		
Hispanic	7	5
Not Hispanic or Latino	30	20
*Hypercholesterolemia*^*a*^		
Negative	20	22
Positive (not on statin)	6	1
Positive (on statin)	4	1

^a^Missing data not available on the internal records or participant refused to provide this information

^18^F-FDG accumulation within the gallbladder was visualized in all the study participants in at least one timepoint during serial imaging (Table 3). No differences were seen in visualization rates between healthy and lymphoma cohorts. No association was detected between GB visualization at each of the study timepoints and different clinical characteristics of the 73 study participants, including age, sex, race, ethnicity, weight, height, BMI, or blood glucose level prior to ^18^F-FDG injection.

**Table 3:** Changes in frequency of gallbladder visualization, uptake intensity, and pattern in respect to serial acquisition timepoints

Gallbladder PET feature	Image acquisition timepoint (min)				
	40	60	90	120	180
*Visualization*					
No	6 (12%)	5 (21%)	1 (2%)	0 (0%)	0 (0%)
**Yes**	**42 (88%)**	**19 (79%)**	**48 (98%)**	**43 (100%)**	**30 (100%)**
*Uptake intensity*^*a*^					
Less than Liver	29 (69%)	15 (79%)	23 (48%)	9 (21%)	7 (23%)
Equal to Liver	13 (31%)	4 (21%)	24 (50%)	31 (72%)	15 (50%)
Higher than Liver	0 (0%)	0 (0%)	1 (2%)	3 (7%)	8 (27%)
*Uptake pattern*^*b*^					
Wall	29 (69%)	5 (26%)	8 (17%)	2 (4%)	0 (0%)
Luminal	9 (21%)	9 (48%)	36 (75%)	36 (84%)	28 (93%)
Luminal & wall	4 (10%)	5 (26%)	4 (8%)	5 (12%)	2 (7%)

The bold row signifies the frequency of GB visualization that was further analyzed regarding other features in subsequent rows

^a^Association between timepoint and gallbladder uptake intensity was statistically highly significant (*P* < 0.0001). Data from scans with gallbladder uptake equal to or higher than the liver activity were summed together

^b^Association between timepoint and uptake pattern was statistically highly significant (*P* < 0.0001). Data labeled as ‘luminal & wall’ were added to their respective categories

Similarly, the actual fasting duration was comparable between participants who demonstrated GB uptake at their first scanning timepoint (40- or 60-min) versus those with more delayed visualization (10.8 ± 3.2 vs. 11.2 ± 3.1 h). Also, there was no significant difference in fasting duration between the healthy group (10.6 ± 2.8 h) and the lymphoma group (11.2 ± 3.8 h).

GB visualization was not linked to injected activity dose, though image interpretation was more challenging in the healthy cohort sub-group that received low-dose ^18^F-FDG injection (N = 15) due to increased image noise, especially for the delayed timepoints. Also, no association was seen with any given history of smoking, diabetes, hyperlipidemia, or cardiovascular, autoimmune, or oncologic conditions. Moreover, the presence of hepatic steatosis showed no significant relationship with GB visualization, uptake intensity or pattern.

### Gallbladder uptake intensity and pattern

**Fig. 2:**
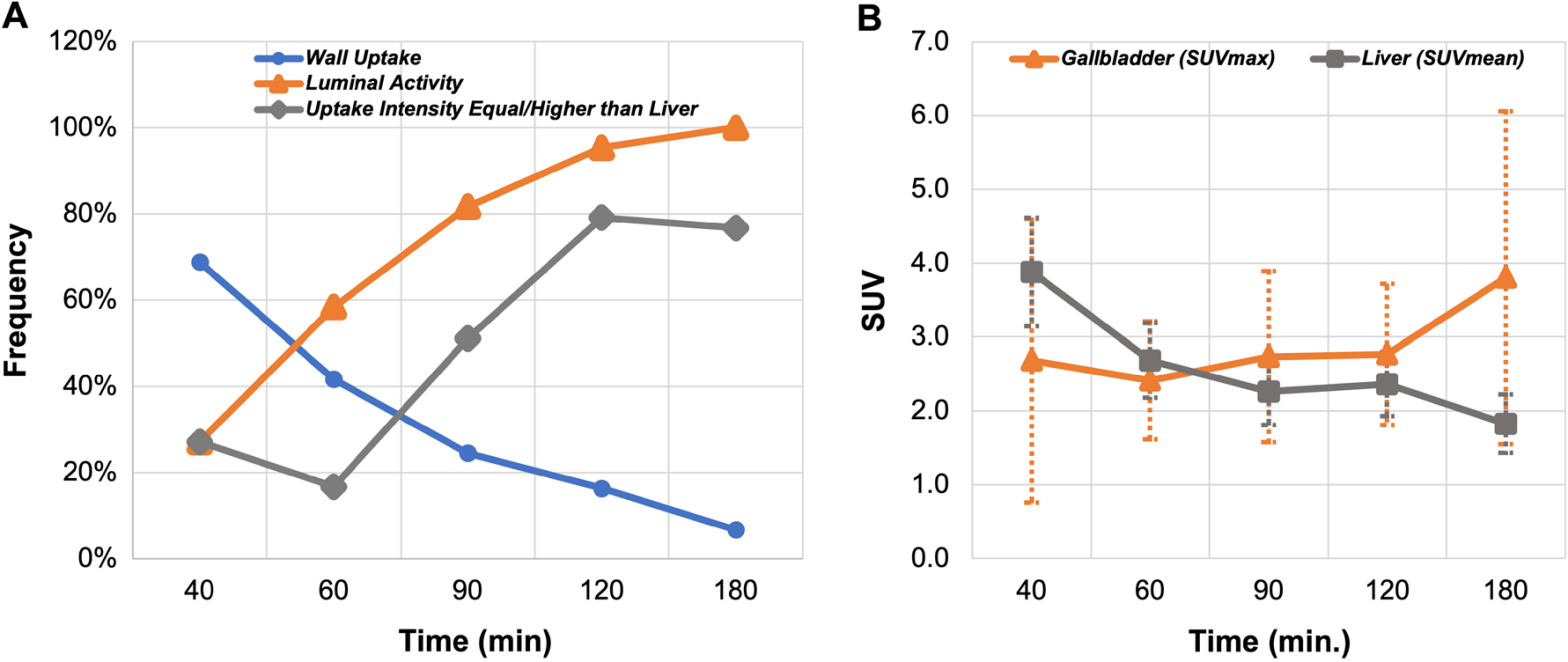
Qualitative and quantitative changes in gallbladder uptake over time. Frequency of gallbladder visualization (wall & lumen) and changes in the uptake intensity in respect to the liver background activity (**A**), and gallbladder & liver standardized uptake values (**B**) in relation to the scanning timepoints in 73 study participants. Error bars represent the standard deviation. Please note that the same subject was not scanned across all the given timepoints. Details of the study subgroups and scanning protocols are summarized in Table 1. Also, please note that the small category where both wall and lumen were visualized was added to their respective category in this graph. Details about the exact numbers are given in Table 3

**Fig. 3:**
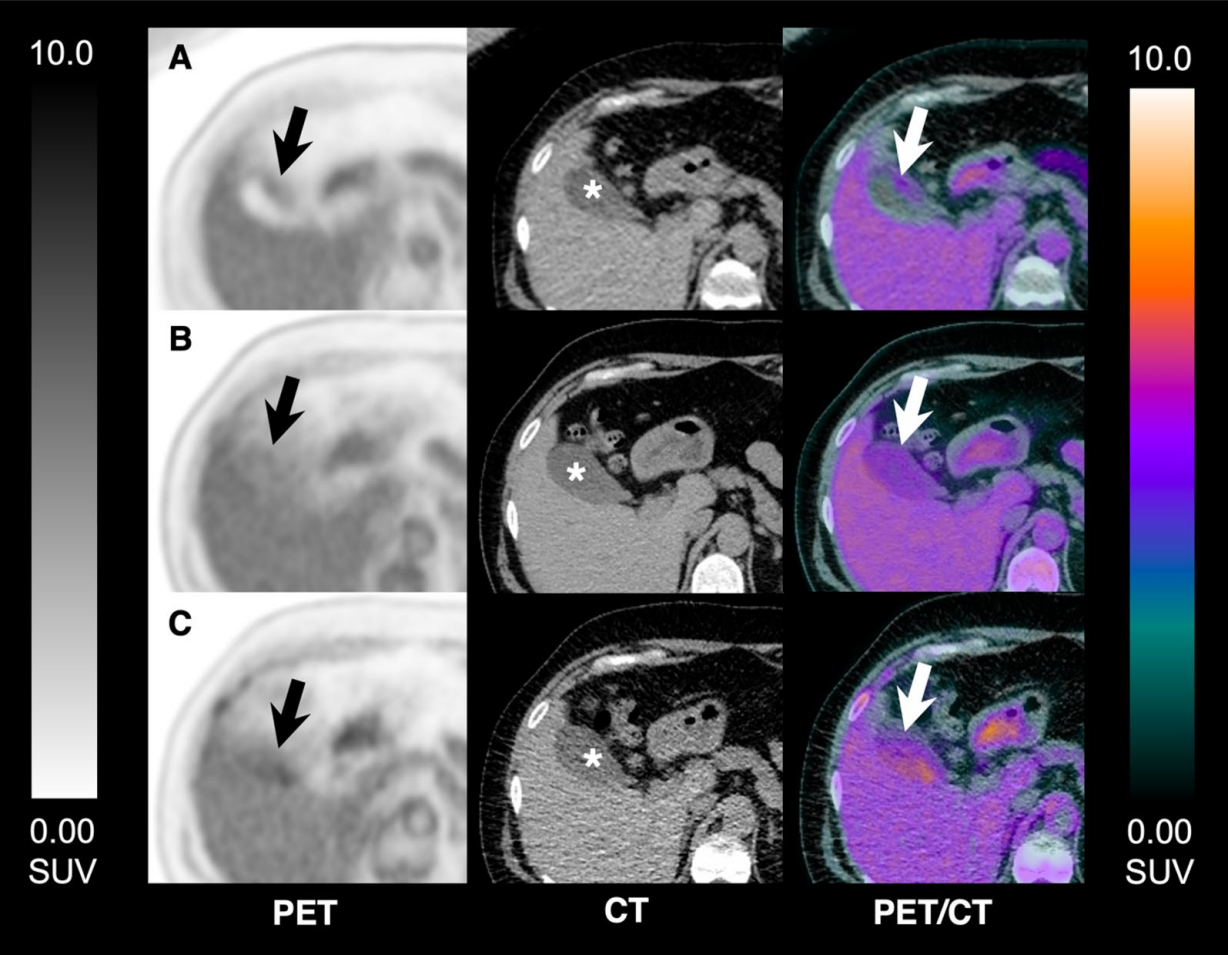
Changes in the gallbladder ^18^F-FDG uptake pattern and intensity on serial total-body PET/CT acquisitions. Axial PET, CT and fused images acquired after i.v. injection of 389 MBq in a 53 y/o healthy participant. Images at 40-min timepoint show wall activity, equal to the liver background; images at 90-min timepoint show luminal uptake, equal to the liver uptake; and images acquired at 180-min post-injection show increased luminal uptake, higher than that of the liver background. Arrows: gallbladder; Asterisks: gallbladder lumen; **A** PET, CT and fused PET/CT images at 40-min timepoint; **B** PET, CT and fused PET/CT images at 90-min timepoint; **C** PET, CT and fused PET/CT images at 180-min timepoint

GB uptake intensity and pattern were strongly associated with the imaging timepoint (Fig. 2). Higher GB uptake intensity with respect to liver background and predominantly luminal localization of tracer activity, rather than GB wall uptake, were seen significantly more frequently (*P* < 0.001) in the delayed timepoints (90–180 min) compared to early timepoints (40–60 min) (Fig. 3).

Quantitatively, 11 subjects were not amenable for accurate quantification due to significant respiratory motion (8 healthy participants and 3 lymphoma patients). In the remaining 62 study participants, the average GB SUVmax values for the 40-, 60-, 90-, 120-, and 180-min timepoints were, respectively, 2.7 ± 1.9, 2.4 ± 0.8, 2.7 ± 1.2, 2.8 ± 1.0, and 3.8 ± 2.3, while the average liver SUVmean for the same timepoints were 3.9 ± 0.7, 2.7 ± 0.5, 2.3 ± 0.5, 2.4 ± 0.4, and 1.8 ± 0.4, respectively.

**Fig. 4:**
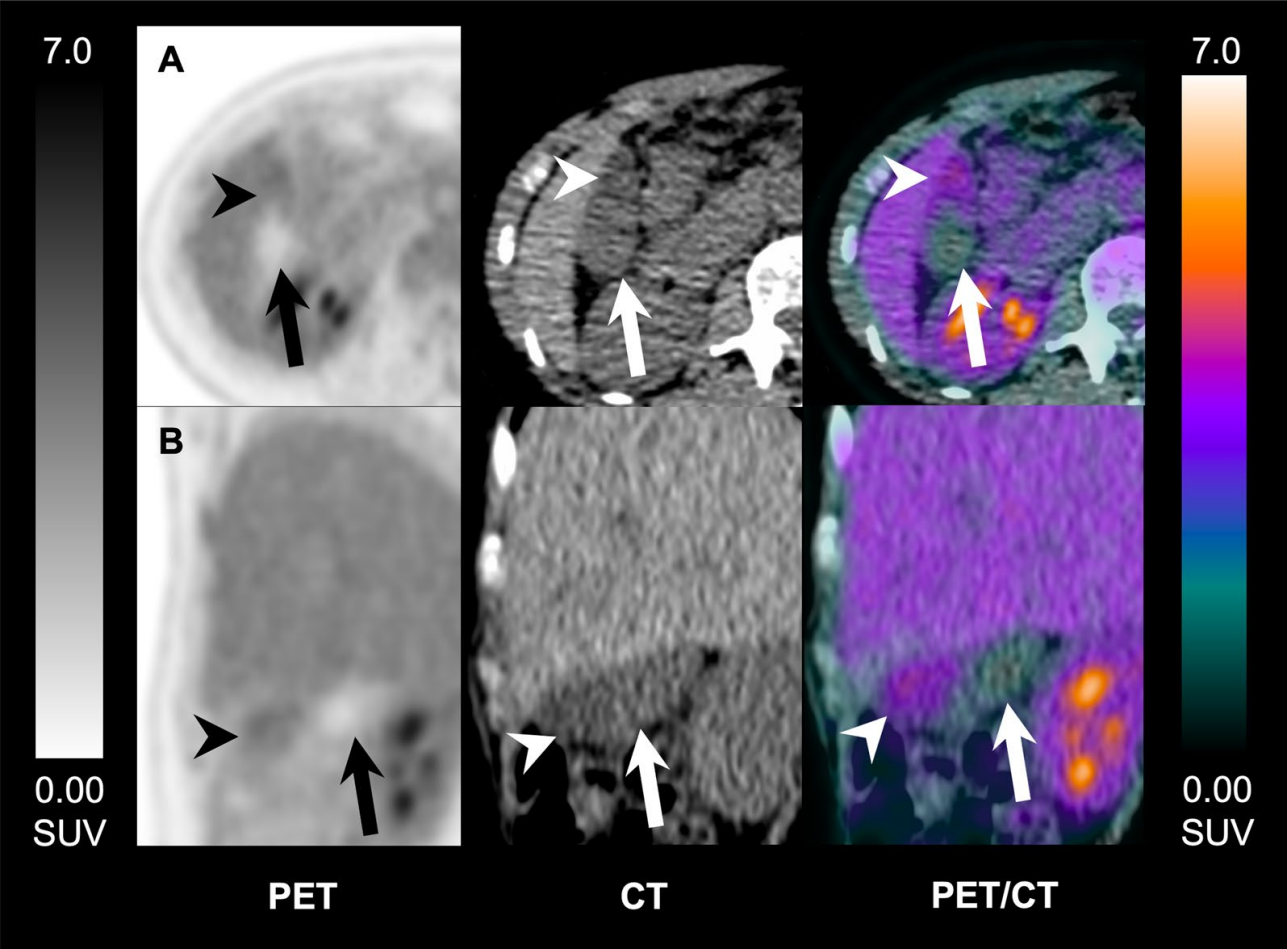
Uneven ^18^F-FDG uptake in the gallbladder. PET, CT and fused PET/CT images of a 61 y/o healthy female participant, scanned at 90-min post-injection of 334 MBq of ^18^F-FDG. Images show distribution of ^18^F-FDG uptake in the gallbladder: one portion shows uptake with lower attenuation on the corresponding CT, while the other portion shows no uptake and higher attenuation on the corresponding CT. These findings suggest the presence of different luminal content. Arrowhead: gallbladder portion showing tracer uptake with lower attenuation on the corresponding CT; arrows: gallbladder portion showing no uptake and higher attenuation on the corresponding CT.** A** axial,** B** coronal, planes of PET, CT and fused PET/CT images

A characteristic GB luminal ^18^F-FDG distribution was observed in 3 patients, where it was visualized in one portion of the GB lumen, while the remaining portion showed no tracer accumulation. On the corresponding CT scans, these two luminal areas showed visually distinct attenuation levels, suggesting different bile composition (Fig. 4). We assume this finding could be related to the presence of biliary sludge: this buildup of biliary compounds sits in the body and neck of the GB, which are the lower parts when the patient is in the supine position, while more water based, non-organized content will lie on top, in the fundus area. This lighter content is more likely to hold the excreted ^18^F-FDG in solution.

It is worth mentioning that no focal or diffuse ^18^F-FDG uptake suggestive of GB abnormalities, e.g., masses or gross wall thickening, was detected in any of the study participants. Incidental gallstones were seen in 2 healthy participants.

## Discussion

In this work, tracer accumulation within the gallbladder was visualized in all the study participants, both healthy participants and lymphoma patients, in at least one timepoint during serial imaging. These findings contradict an earlier study that reported detection rate less than 1% [5] and could relate to the characteristics of new generation total-body PET/CT scanners, which provide images with greater spatial resolution and signal-to-noise ratio compared to prior generations of PET/CT scanners [8, 12, 14, 17]. Interestingly, GB was still systematically visualized, even in the subgroup that received 1/20^th^ of the standard injection dose, suggesting that spatial resolution is a key factor for this application.

Imaging protocols employing total-body PET can be expected to demonstrate findings that may not have been previously visualized or described before (e.g., blanching defects [18]). Recent works carried out using total-body PET/CT demonstrated that SUVs in small structures, such as walls of major blood vessels, are higher than that usually seen from standard scanners [19]. Also, uptake in normal physiologic structures becomes apparent or more prominent than what is reported on conventional scanners, such as in the adrenal glands, the pituitary gland, and grey matter of the spinal cord [17, 20].

We hypothesize that the described ^18^F-FDG distribution in the gallbladder in this study to be physiologic. Two major observations that backup this assumption are: first, the GB visualization was systematic and seen invariably across healthy and lymphoma patients, at least in one scanning timepoint. Second, a significant positive association was observed between luminal tracer build-up and scanning timepoint, supporting the idea that ^18^F-FDG accumulates inside the gallbladder over time, until it gets excreted by physiological gallbladder emptying.

We described the presence of not only luminal activity but also wall uptake, the latter at a much higher frequency in the early timepoints (40- & 60-min). While we do not hold a clear explanation of the anatomic/histologic structure within the gallbladder that is responsible for wall uptake, and why it tends to disappear at later timepoints, we hypothesize it might be related to smooth muscle uptake, which might be more prominent in the earlier phases after tracer injection. This could be, at least partially, attributed to early local blood flow and late physiologic washout. This assumption might be supported by the higher frequency of early tracer detection in the wall (69%, 26%, 17%, 5% and 0% at 40-, 60-, 90-, 120-, and 180-min timepoints, respectively). However, this hypothesis needs further validation, for example through kinetic modeling and parametric imaging [21].

Our data are partially consistent with another retrospective study [6], in which a small, 12-patient cohort was scanned at two timepoints (mean: 73- and 142-min post-injection) to assess changes in uptake intensity and pattern over time. The authors reported absence of ^18^F-FDG in the gallbladder at the first time point in 11/12 patients, while uptake was observed in 8/12 patients at the delayed time point. These authors were the first to report ^18^F-FDG accumulation within the lumen, rather than wall, of non-pathologic gallbladder and suggested that this phenomenon may occur as a result of physiologic ^18^F-FDG excretion into the bile.

Similarly, in our work we noticed increasing^18^F-FDG luminal accumulation at later timepoints. The underlying physiologic mechanism could be explained as follows. Normal bile includes glucose in negligible amounts due to active reabsorption at the peripheral (cholangiocytes) end of the hepatic lobule after being secreted at the bile canaliculi by hepatocytes [22]. While passing through the hepatocytes, both glucose and ^18^F-FDG initially enter canalicular bile through energy independent transport mechanism (glucose transporter 2, GLUT2); however, glucose, but not ^18^F-FDG, is subsequently reabsorbed from bile by cholangiocytes. Glucose is transported from bile to blood via sodium glucose transporter 1 (SGLT-1), expressed on the cholangiocytes apical plasma membrane, then by GLUT-1 transporters expressed on the basolateral plasma membrane [23]. Because fluoride replaces the hydroxyl group on Carbon 2 of the FDG molecule, ^18^F-2-FDG is not transported by SGLTs in the cholangiocytes [24, 25]. Accordingly, ^18^F-FDG ends up in the gallbladder instead of being reabsorbed back into circulation. These expectedly minute quantities of ^18^F-FDG may not be amenable to detection by conventional scanners using standard imaging protocols. However, performing the scan on high-sensitivity scanner, together with delayed images acquired for full 20 min, allowed for consistent visualization of the activity. It is worth mentioning that we were not able to detect any increase in ^18^F-FDG activity within the small intestine related to GB luminal uptake and expected subsequent biliary-to-bowel transit. This is probably because diffuse low-grade bowel uptake is part of normal ^18^F-FDG biodistribution, and numerous physiologic and pathologic conditions can significantly alter this distribution [26, 27]. Accordingly, we assume the contribution from the ^18^F-FDG within the excreted bile might be too small to be noticed, especially when considering the physiologic bowel peristalsis.

Our study has several strengths, including the use of an ultrasensitive high-resolution total-body PET/CT scanner, the employment of multiple sequential timepoints, inclusion of a healthy cohort, and the enrichment of our dataset with a group with diverse ethnic/racial backgrounds. On the other hand, we recognize several limitations that affected our study. First, our findings were not independently validated by correlation with a GB-related questionnaire, other imaging modalities (e.g., abdominal ultrasound), or histopathology. Additionally, several participants were scanned on outpatient-basis and their full medical record was not available at our institution. Second, we frequently encountered mismatch between the PET and CT due to respiratory motion which could affect the accuracy of quantification [28]; accordingly, we relied mainly on the qualitative description in respect to physiologic liver uptake. The supplied quantitative analyses should be cautiously interpreted. Work towards the development of total-body motion correction is ongoing [29, 30]. Third, we employed non-contrast low-dose or ultra-low-dose CT protocols, which were adequate for anatomical localization and attenuation correction but were harder to leverage further (e.g., for quantitively evaluating the serial changes in GB volumes). Lastly, this is an exploratory work with limited number of participants, which might not have enough power to detect small differences between or within subgroups. However, the uniform visualization of gallbladder could be used to inform future studies intended to investigate further pathophysiologic aspects, for example, how the gallbladder uptake would change before and after specific systemic treatment in oncologic subjects.

## Conclusions

Our study demonstrated visualization of the gallbladder in all healthy subjects and lymphoma patients in ^18^F-FDG scans performed at 120 and 180 –minutes post-injection, when imaged on a high-sensitivity high-resolution total-body PET/CT scanner. The tracer uptake was more evident in the gallbladder wall at earlier timepoint scans, whereas luminal activity was more prevalent in delayed images with tracer accumulation increasing with time. We believe that the consistent visualization of GB can be described as a physiologic finding and should not be mistaken for pathology or uncommon finding.

## Data Availability

The datasets used and/or analyzed during the current study are available from the corresponding author on reasonable request.

## Abbreviations

LAFOV: Long-axial field-of-view
^18^F-FDG: ^18^F-2-fluorodeoxyglucose
PET/CT: Positron emission tomography/computed tomography
GB: Gallbladder
TB: Total-body
IRB: Institutional Review Board
EMR: Electronic Medical Record
OSEM: Ordered subset expectation maximization
TOF: Time-of-flight
PSF: Point-spread function
VOIs: Volumes of interest
HU: Hounsfield Unit
BMI: Body-mass index
